# Recurrent atelectasis and brain infarction in a patient with anti-neutrophil antibody negative eosinophilic granulomatosis with polyangiitis: a case report

**DOI:** 10.1186/s41927-021-00200-8

**Published:** 2021-09-01

**Authors:** Ken-ei Sada, Atsushi Miyauchi, Daisuke Hashimoto, Riku Ino, Shigeru Nojima, Shingo Yamanaka, Masafumi Kawamura

**Affiliations:** 1 Department of Internal Medicine, Kochi Prefectural Hata-Kenmin Hospital, 3-1Yoshina, Yamanachou, Sukumo, 788-0785 Japan; 2 Department of Clinical Epidemiology, Kochi Medical School, Kochi University, Nankoku, Japan

**Keywords:** Eosinophilic granulomatosis with polyangiitis, Atelectasis, Brain infarction, Eosinophil, Mepolizumab

## Abstract

**Background:**

Eosinophilic granulomatosis with polyangiitis (EGPA) is an anti-neutrophil antibody (ANCA)-associated necrotizing vasculitis, which predominantly affects small to medium vessels, and is associated with asthma and eosinophilia. EGPA has two different pathogenic aspects: eosinophilic granulomatous inflammation and ANCA-associated inflammation. A recent histological study of peripheral nerves showed that not only ANCA-associated inflammation but also eosinophil-associated vascular occlusion leads to ischemia. Endobronchial involvement is relatively common especially in the patients with granulomatosis with polyangiitis but rare in patients with EGPA. Central nervous system (CNS) involvement is also rare in patients with EGPA, the pathogenesis and relationship between these two rare conditions have not been elucidated.

**Case presentation:**

A 62-year-old woman was admitted with numbness, purpura, and eosinophilia. She had a 3-year-history of bronchial asthma. Chest computed tomography showed left lower lobe collapse, and brain magnetic resonance imaging indicated occipital lobe infarction. Skin biopsy findings led to the diagnosis of EGPA. ANCA test results were negative. All symptoms improved after initiating glucocorticoids. However, atelectasis and brain infarction relapsed with increasing eosinophil counts. Atelectasis quickly disappeared with increasing glucocorticoid dose, and glucocorticoid could be reduced to a maintenance dose after the initiation of mepolizumab.

**Conclusion:**

Both atelectasis and brain infarction might develop not only via ANCA-associated inflammation but also via eosinophilic inflammation.

## Background

Eosinophilic granulomatosis with polyangiitis (EGPA) is an anti-neutrophil antibody (ANCA)-associated vasculitis, which is a necrotizing vasculitis that predominantly affects small to medium vessels, and is associated with asthma and eosinophilia [1]. EGPA has two different pathogenic aspects: eosinophilic granulomatous inflammation and ANCA-associated inflammation. A recent histological study of peripheral nerves showed that not only ANCA-associated inflammation but also eosinophil-associated vascular occlusion leads to ischemia [2].

Lung and nervous system involvement is a common manifestation in patients with EGPA [3]. The most frequent manifestations of lung and nervous system involvement are peripheral nerve disorder and lung infiltration, respectively. Endobronchial involvement is relatively common in patients with granulomatosis with polyangiitis but rare in patients with EGPA [4, 5]. Regarding nervous system involvement, only 5% of patients with EGPA developed central nervous system (CNS) involvement [6]. The pathogenesis and relationship between these two rare conditions in EGPA have not been elucidated.

Here, we report a patient with ANCA negative EGPA, who developed recurrent atelectasis and brain infarction. Mepolizumab helped reduce glucocorticoid dosage without relapse.

## Case presentation

A 62-year-old woman developed bronchial asthma in 2015. She had no history of smoking. She had never been diagnosed with dyslipidemia, diabetes, or hypertension before. She experienced atelectasis in May 2018, but it resolved spontaneously. In August 2018, she developed leg edema and purpura with eosinophilia and was referred to our hospital.

**Fig. 1:**
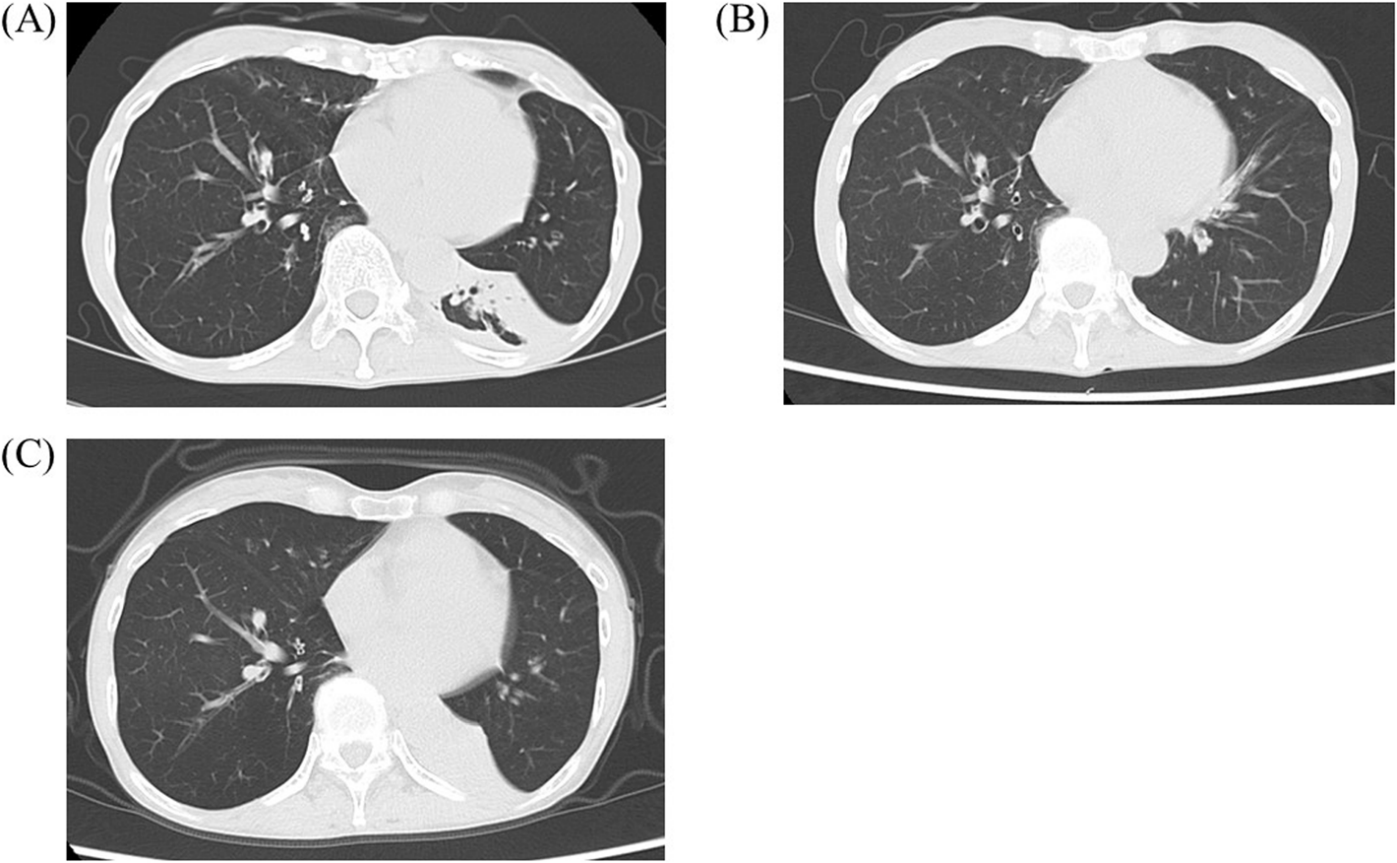
Chest computed tomography. **a**: Before treatment; **b**: 7 days after the initial treatment; and (**c**): 20 months after the initial treatment

**Fig. 2:**
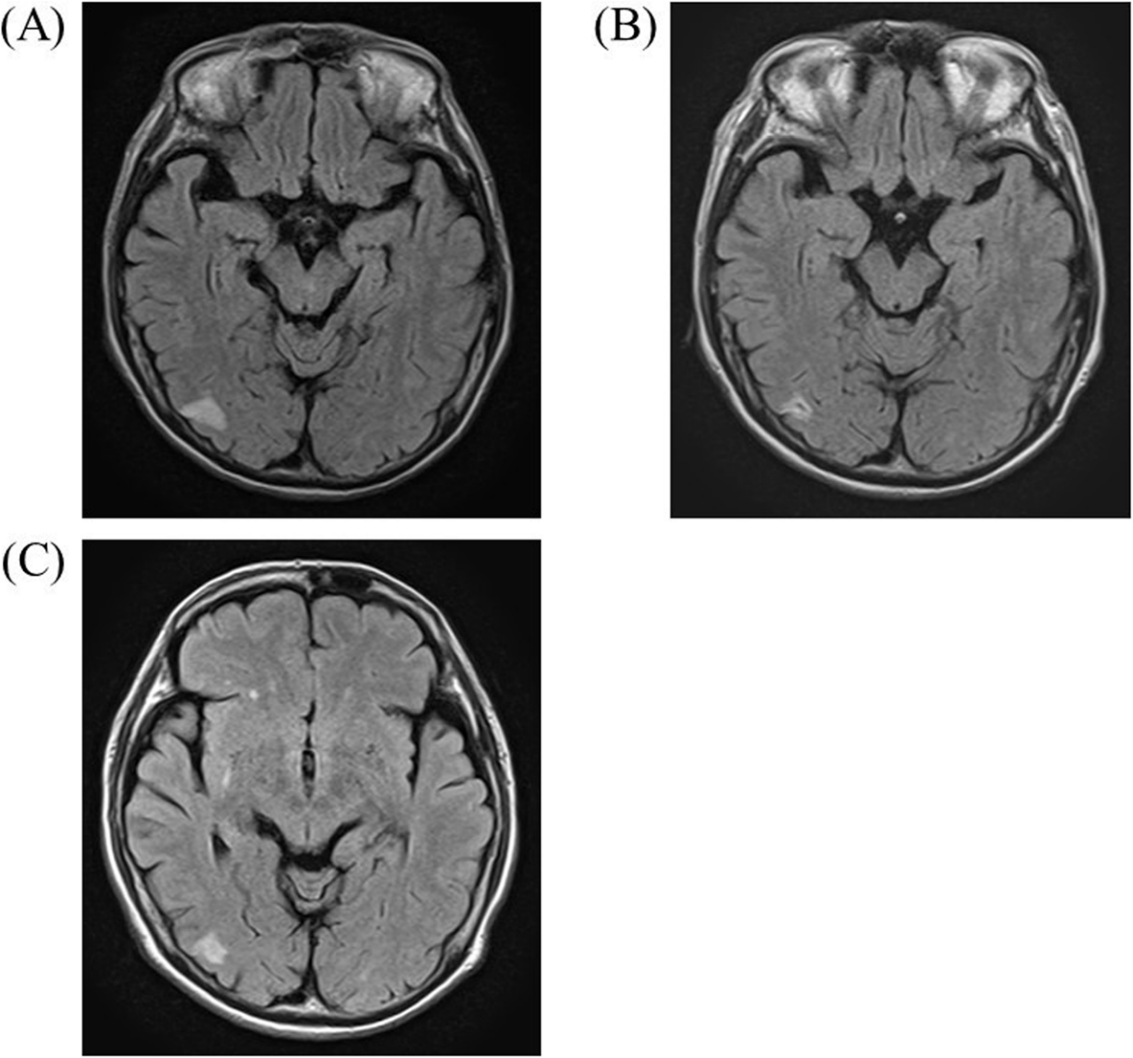
Brain magnetic resonance imaging. **a**: Before treatment, **b**: 4 months after the initial treatment, (**c**): 16 months after the initial treatment

On admission, she complained of numbness in her distal extremities. She was alert. Findings of the physical examination were as follows: height, 163 cm; weight, 41 kg; body temperature, 36.8 °C; blood pressure, 125/84 mmHg; and O_2_ saturation in room air, 95%. Diminished breathing sounds were noted in the left upper back, with no abnormal findings in the head, neck, or abdomen. Pitting edema and a few palpable purpuras were detected in both lower legs. Neurological examination revealed peripheral sensory neuropathy in the lower extremities but no CNS disorder. The laboratory values were as follows: white blood cells, 43,400/μL (eosinophil count, 36,890/μL); platelets, 26.5 × 10^4^/μL; and C-reactive protein, 1.31 mg/dL (normal range < 0.15 mg/dL). Protein uria, hematulia, or any other abnormal findings were not found in urinalysis. Rheumatoid factor was 72 IU/mL (normal range < 15 IU/mL); however, test for proteinase 3-antineutrophil cytoplasmic antibodies and myeloperoxidase- ANCAs were negative. Ultrasound echocardiography showed no impairment of cardiac function. Chest computed tomography (CT) showed left lower lobe collapse (Fig. 1a). Diffusion-weighted imaging (DWI) and fluid-attenuated inversion recovery (FLAIR) of the brain magnetic resonance imaging (MRI) showed a high-intensity area in the occipital lobe, which indicated subacute brain infarction (Fig. 2a). A biopsy of a skin sample taken from the purpura in the lower leg revealed leukocytoclastic vasculitis and eosinophil filtrations around the blood vessels. The patient was finally diagnosed with EGPA based on physical, laboratory, and histological findings.

After the initiation of 40 mg/day of prednisolone (PSL), her numbness and purpura quickly improved. Eosinophil count also decreased to 13,450/μL on day 7 after initiating PSL. Chest CT on day 7 also showed the disappearance of lung collapse (Fig. 1b). In January 2019, brain MRI showed high intensity, indicating an old ischemic change (Fig. 2b).

**Fig. 3:**
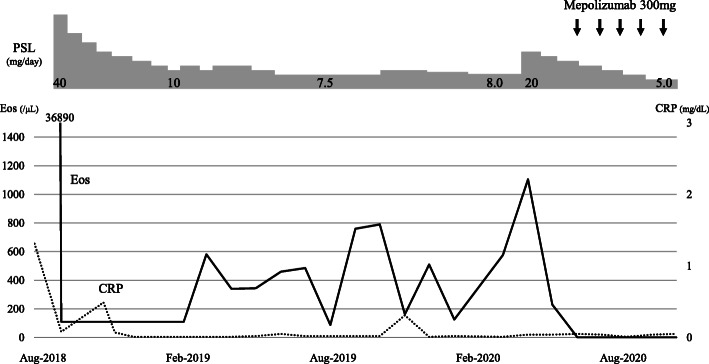
Clinical course. CRP, C-reactive protein; Eos, eosinophil; PSL, prednisolone

The clinical course of this patient is shown in Fig. 3. Although the patient remained symptom free, it was difficult to reduce the PSL dose without an eosinophil increase. In January 2020, a high-intensity area near the previous lesion emerged in FLAIR but not in DWI without any symptoms (Fig. 2c). With an increase in eosinophil count to 1105/μL with a PSL dose of 8.0 mg/day in April 2020, chest CT showed recurrence of atelectasis without any symptoms (Fig. 1c). After increasing to 20 mg/day of PSL, atelectasis quickly disappeared on chest radiography. Follow-up brain MRI showed no new abnormal lesion in July 2020.

After the initiation of mepolizumab (300 mg/4 weeks), the PSL dose was reduced to 5 mg/day, and remission was maintained.

## Discussion and conclusions

To the best of our knowledge, this is the first case of a patient with EGPA presenting with both recurrent atelectasis and recurrent cerebral infarction. We differentially diagnose EGPA from hypereosinophilic syndrome by the histological confirmation of vasculitis.

Lung atelectasis may occur because of eosinophilic endobronchial lesions. Endobronchial disease is relatively common in the patients with ANCA-associated vasculitis, especially granulomatosis with polyangiitis [7]. It is well known that common CT findings of EGPA include subpleural ground-glass opacity or consolidation with a lobular distribution, centrilobular nodules, bronchial wall thickening, and interlobular septal thickening [8]. Only one case series reported that one of the seven patients with EGPA showed atelectasis [4]. Recently, one EGPA case with endobronchial mucosal lesions reportedly exhibited atelectasis, and biopsy of the mucosal lesions showed extensive necrosis and hemorrhage with a large number of eosinophil infiltrates [5]. Our case showed recurrence of atelectasis with an increasing eosinophil count. Atelectasis develops in patients with bronchocentric granulomatosis, which is an eosinophilic lung disease. Although EGPA has two different pathogenic aspects: eosinophilic inflammation and ANCA-associated inflammation, endobronchial lesions might be caused not only by ANCA-associated inflammation but also by eosinophilic inflammation in patients with EGPA.

Ischemic cerebrovascular lesions could develop even in ANCA-negative patients. CNS involvement is a rare condition in patients with EGPA. A previous report showed that 5% of patients with EGPA developed CNS involvement. Another report showed that 52% of patients with EGPA with CNS involvement had ischemic cerebrovascular lesion and 48% of patients with ischemic cerebrovascular lesion cases among patients were ANCA negative [9]. Recently, a histological study of peripheral nerves showed that eosinophil-associated vascular occlusion leads to ischemia [2]. Our present case was ANCA negative with an ischemic cerebrovascular lesion, and the patient experienced recurrence with an increasing eosinophil count. Therefore, eosinophilic inflammation might lead to cerebrovascular ischemia in patients with ANCA-negative EGPA.

In conclusion, atelectasis and cerebral infarction might develop via eosinophilic inflammation in patients with EGPA. Mepolizumab might be an optimal treatment option for refractory patients with eosinophilic inflammation.

## Data Availability

Not applicable.

## Abbreviations

EGPA: Eosinophilic granulomatosis with polyangiitis
ANCA: Anti-neutrophil antibody
CNS: Central nervous system
CT: Computed tomography
DWI: Diffusion-weighted imaging
FLAIR: Fluid-attenuated inversion recovery
MRI: Magnetic resonance imaging
PSL: Prednisolone
